# Comparison of Ruminal Degradability, Indigestible Neutral Detergent Fiber, and Total-Tract Digestibility of Three Main Crop Straws with Alfalfa Hay and Corn Silage

**DOI:** 10.3390/ani11113218

**Published:** 2021-11-11

**Authors:** Erdan Wang, Jidong Wang, Jiaying Lv, Xiaoge Sun, Fanlin Kong, Shuo Wang, Yajing Wang, Hongjian Yang, Zhijun Cao, Shengli Li, Wei Wang

**Affiliations:** Beijing Engineering Technology Research Center of Raw Milk Quality and Safety Control, State Key Laboratory of Animal Nutrition, College of Animal Science and Technology, China Agricultural University, No. 2 Yuanmingyuan West Road, Haidian, Beijing 100193, China; wangerdan@cau.edu.cn (E.W.); SY20203040696@cau.edu.cn (J.W.); b20183040337@cau.edu.cn (J.L.); B20193040347@cau.edu.cn (X.S.); fanlinkong@cau.edu.cn (F.K.); b20213040351@cau.edu.cn (S.W.); yajingwang@cau.edu.cn (Y.W.); yang_hongjian@sina.com (H.Y.); caozhijun@cau.edu.cn (Z.C.); lisheng0677@163.com (S.L.)

**Keywords:** main crop straws, ruminal degradability, indigestible neutral detergent fiber, total-tract digestibility

## Abstract

**Simple Summary:**

Corn straw (*Zea mays*, CS), rice straw (*Oryza sativa*, RS), and wheat straw (*Triticum aestivum*, WS) are the three main crop straws worldwide. Few studies on indigestible neutral detergent fiber (iNDF) and total-tract digestibility (TTD) of crude protein (CP), neutral detergent fiber (NDF), and acid detergent fiber (ADF) of these crop straws are available, which limits their utilization in dairy diets. Here, we compared the ruminal degradability, iNDF_288_ content, intestinal digestibility, and TTD for the CP, NDF, and ADF of these three crop straws with alfalfa hay (*Medicago sativa*, AH) and corn silage (*Zea mays*, CSil). The results showed that CS, RS, and WS had higher ruminal potential NDF degradation, intestinal digestible CP, and lower iNDF_288_ content compared to AH. Greater accuracies for regression equations capable of predicting the iNDF_288_ content and TTD were also generated based on chemical composition and ruminal degradation kinetics. Incorporating this information into rations could improve our ability to optimize the utilization of main crop straws in balanced dairy diets.

**Abstract:**

Three main crop straws including corn straw (*Zea mays*, CS), rice straw (*Oryza sativa*, RS), and wheat straw (*Triticum aestivum*, WS), and two forages including alfalfa hay (*Medicago sativa*, AH) and corn silage (*Zea mays*, CSil) were analyzed in order to compare their ruminal degradability, indigestible neutral detergent fiber (iNDF), intestinal digestibility (ID), and their total-tract digestibility (TTD) of crude protein (CP), neutral detergent fiber (NDF), and acid detergent fiber (ADF) using both an in situ nylon bag technique and a mobile nylon bag technique. The forage samples were incubated in the rumen for 6, 12, 16, 24, 36, 48, 72, and 288 h, respectively, to determine their ruminal degradability. Prior to intestinal incubation, forage samples were incubated in the rumen for 12 h and 24 h to determine the ruminal degradable content of CP, NDF, and ADF, respectively, and for 288 h to determine their iNDF_288_ content. Residues from the ruminal undegradable fractions (12 h for CP, 24 h for NDF and ADF) were subsequently inserted into the duodenum through a cannula to determine their intestinal digestible content. Here, the TTD of CP, NDF, and ADF were determined as the ruminal degradable content + intestinal digestible content. The results showed that AH had the highest iNDF_2.4_ (calculated as acid detergent lignin content × 2.4) and iNDF_288_ values (379.42 and 473.40 g/kg of NDF), while CS and CSil had the lowest iNDF_2.4_ values (177.44 and 179.43 g/kg of NDF). The ruminal degradability of CP, NDF, and ADF for CS, RS, and WS were lower than those of AH and Csil during the first 48 h of incubation. The potential degradation fraction of CP, NDF, and ADF for CSil was the highest; CS, RS, and WS were intermediate; and AH was the lowest (*p* < 0.05). CS, RS, and WS had a lower intestinal digestibility with respect to their rumen undegradable content of NDF (*p* < 0.05), and lower TTD of CP, NDF, and ADF (*p* < 0.05) compared to AH and CSil. General regression equations with satisfactory accuracy (R^2^ ≥ 0.828) were derived to predict iNDF_288_ and TTD based on their chemical compositions and the ruminal degradation kinetics of different forages. Incorporating this information into rations could improve our ability to optimize main crop straws utilization and milk production.

## 1. Introduction

Corn straw (*Zea mays*, CS), rice straw (*Oryza sativa*, RS), and wheat straw (*Triticum aestivum*, WS) are the main crop straws due to the fact that corn, wheat, and rice are the main grain crops consumed worldwide (with a production of 1125, 775.8, and 505 million metric tons, respectively, in 2020–2021) [1]. Particularly, China generates over 600 million tons of crop straws annually, of which CS, RS, and WS make up the top three components of the national yield [2,3,4,5]. With a national utilization rate of 80% in 2015, there is still a large amount of crop straw that is burned in open fields, resulting in not only environmental pollution but also underutilization of biomass resources [3].

Rumen depends on rumen microorganisms, which represent one of the most elegant and efficient cellulose-digesting systems in nature [6,7,8]. Ruminants can convert low-value plant biomass into high-value animal protein including milk, meat, and fiber products, while also releasing methane (CH_4_), the single largest anthropogenic greenhouse gas [9,10]. However, the nutritional value of crop straws was reported to be low due to their low contents of crude protein (CP), metabolizable energy (ME), minerals, and vitamins, and high contents of polysaccharides, lignin, and silica content, which may restrict their use as feed for ruminants [11,12,13]. Moreover, when CS is fed as the main forage in diets, the production performance of livestock is always compromised [13,14]. The lactational performance of dairy cows and the total-tract apparent digestibility of all the nutrients significantly decreased when replacing the alfalfa hay (AH) with corn stover and RS as the main forage (30% of DM diet) of isonitrogenous diets [14]. On the other hand, reducing the corn stover dosage to 19% of the dietary DM produced no significant differences in dry matter intake (DMI) and milk production [15]. Notably, similar milk yield and total-tract digestibility (TTD) were observed when the dairy cows were fed equivalent concentrations of neutral detergent fiber (NDF) from corn silage (CSil), AH, WS, and corn stover (50.8–101 g/kg of diet DM) [12]. Different forages vary in their digestibility of NDF, which is the most important trait in feed value determinations, and dominate the variability in total diet digestibility, helping in predicting DMI and lactating performance of dairy cows [16,17,18]. Meanwhile, forage is important in ruminant nutrition, not only as a source of ME but also contains sufficient physically effective neutral detergent fiber (peNDF) to stimulate rumination and saliva production, which buffers the rumen and promotes rumen health [19].

Digestibility is the most important trait in feed value determinations of forages [17]. The in situ nylon bag technique (ISNBT) and the mobile nylon bag technique (MNBT) are frequently used to predict the degradability and digestibility of nutrients and feeding value of feeds for animal production systems. The indigestible NDF (iNDF_288_), determined by a 288 h ruminal in situ incubation, has been demonstrated to be a good predictor of forage digestibility and is an important parameter in mechanistic rumen models [17,19,20,21,22]. Formulating a diet to a specific level of NDF without reference to the iNDF could markedly affect its resulting intake, digestibility, and ME content [19]. Lippke et al. [23] suggested that maximum iNDF consumption is about 20 g/kgBW^0.75^ per day. However, more research is required to resolve if this value is relevant for different production systems and different forages [19]. The Cornell Net Carbohydrate and Protein System (CNCPS) [24] and Cornell-Penn-Miner (CPM) Dairy [25] estimate the iNDF in forages using the formula ADL × 2.4 (iNDF_2.4_). However, tropical (C4) forages have large variations in iNDF and do vary in ruminal degradability. Total-tract NDF digestibility (TTNDFD), calculated as ruminal NDF digestion + hindgut NDF digestion, has recently been demonstrated to be a good predictor of forage digestibility [26,27]. A large number of studies have been conducted to investigate the ruminal degradability, iNDF, and total-tract digestibility (TTD) of concentrate feeds [20,28,29,30,31,32,33,34,35,36,37,38,39,40,41] and high-quality forages such as CSil [26,41,42,43,44,45,46,47,48,49], AH [41,42,46,48,49,50], and oat hay [46,48] during the last two decades. However, studies on ruminal degradability, iNDF content, intestinal digestibility (ID), and TTD of the crop straws, especially CS, RS, and WS are limited. Sarnklong et al. gave an overview of the availability, nutritive quality, and possible strategies to improve the utilization of RS as a feed ingredient for ruminants [11].

Better assessment and awareness amongst nutritionists on the importance of iNDF in crop straws will improve the capacity of nutritionists to predict NDF digestibility and therefore most effectively develop balanced diets. Therefore, the objectives of this study were to (1) characterize the ruminal degradability, iNDF_288_ content, ID, and TTD of CP, NDF, and ADF of three main crop straws (CS, RS, and WS) and (2) to compare these parameters with those of AH and CSil using the ISNBT and MNBT techniques.

## 2. Materials and Methods

Feeding and management of cows used in this study were according to the China Agriculture University animal research committee protocol (Protocol number: 2013-5-LZ). This experimental protocol was approved by the Institutional Animal Care and Use Committee of China Agricultural University (Protocol number: AW61110202-2; Date: 25 August 2019).

### 2.1. Sample Preparations

One sample for each of the forages was collected in the present study. CS, AH, and CSil samples were collected from Jinyindao dairy farm (Beijing, China), RS and WS were collected from Benniu (Harbin, China) and Huahuaniu (Zhengzhou, China) dairy farms, respectively. CSil and CS were selected as tropical (C4) forages, whereas, RS, WS, and AH belonged to subtropical (C3) forages. All forage samples were dried in an air oven at 65 °C for 48 h, then ground to pass through a 2.5 mm sieve. The chemical composition of the selected forage samples is shown in Table 1.

### 2.2. Cow Management

Three second-parity lactating Holstein dairy cows (days in milk: 196.67 ± 6.35 d; dry matter intake: 23.68 ± 0.88 kg/d; daily milk yield: 28.13 ± 2.35 kg/d; body weight: 648 ± 29 kg) fitted with three-site (rumen, anterior duodenum, and terminal ileum)-cannula (10 cm internal diameter ruminal fistula; T-shaped intestinal fistula; Anscitech Farming Technology Co., Ltd., Wuhan, China) were used for the ruminal and intestinal incubation. Cows were milked and fed three times per day and allowed access to feed and fresh water *ad libitum*. The TMR diet (25.1% corn silage, 11.8% alfalfa hay, 2.0% oat hay, 61.1% concentrate mix; DM basis) with a measured nutrient composition of 25.8% starch, 17.1% CP, 29.7% NDF, and 4.9% ether extract was formulated to meet NRC requirements [51] of a dairy cow producing 31 kg/d milk. Individual feed intake was measured during the study by a roughage intake control system (RIC, Zhenghong Co., Ltd., Shanghai, China).

### 2.3. In Situ Ruminal Incubation and iNDF_288_ Determination

The in situ incubation procedure followed a proposal for a standardized method for forage ingredients [51,52]. Approximately 5 g of samples were filled into heat-sealed nylon bags (8 × 12 cm bag size; 50 μm pore size; sample size to nylon bag surface was about 26 mg/cm^2^ calculated according to Diao et al. [53]) in six repetitions. Each cow was regarded as a replicate, each forage three replicates, and each forage had two parallel replicates per cow (*n* = 6). A maximum of six bags were attached to 50 cm semi-flexible stalks that retained bags within the liquid phase of the ruminal content. Five forages were incubated sequentially in different batches. A total of 270 bags (54 bags per forage) were prepared for in situ incubation. All bags were incubated at the same time of the day (0730 h) before the morning feeding and retrieved according to their respective incubation times. Two duplicated bags per forage were incubated in each cow and simultaneously retrieved at 0 (not incubated in the rumen), 6, 12, 16, 24, 36, 48, 72, and 288 h after incubation [22,51]. The forage samples were ruminally incubated for 12 h to determine the ruminal degradable CP (RDP), 24 h for ruminal degradable NDF (RD_NDF_) and ADF (RD_ADF_) [47], and 288 h to determine iNDF_288_ [22,44,54]. The NDF content in the 288 h residue is normally referred to as the truly indigestible NDF (iNDF_288_) [17,20]. Once retrieved from rumen at each time point, the bags were rinsed and manipulated in cold water until the water turned clear, then dried to a constant weight at 65 °C for 48 h. The dried residues were ground through a 1 mm sieve for further use.

### 2.4. Pretreatment, Intestinal Incubation, and Total-Tract Digestibility Determination

The mobile nylon bag incubation procedure followed the protocol proposed by Kaitho et al. [55]. In the present study, the rumen passage rate (kp) for CP was calculated to be 4.18 according to the prediction models recommended by NRC (2001) [51]. The ruminal incubated residues (12 h for CP, 24 h for NDF and ADF) were ground through a 1 mm sieve and weighed at a rate of 0.5 g per mobile nylon bag (3 × 6 cm bag size; 25 μm pore size) which was subsequently heat-sealed. Twelve duplicated bags per forage were placed in a shaking bath filled with pepsin/HCl solution for 1 h at 39 °C to simulate abomasal digestion before intestinal incubation. Pepsin/HCl solution was prepared by dissolving 1 g of pepsin powder (activity 1:10,000, Beijing Aoboxing Biotech Co., Ltd., Beijing, China) in 1 L of 0.01 mol/L HCl. The three Holstein cows fitted with three-site (rumen, anterior duodenum, and terminal ileum)-cannula were used for the intestinal incubation. Each cow was regarded as a replicate, each forage had three replicates, and each forage had 4 parallel replicates per cow (*n* = 12). Four bags of each forage were individually inserted in random order into the anterior duodenum cannula of each cow at a rate of two bags every 30 min starting from the morning feeding. A maximum of 12 bags can be inserted into the duodenum cannula per cow per day. Bags were collected in the feces from 8 h after incubation. The recovered bags were rinsed and manipulated in cold water until the water ran clear, then dried to a constant weight at 65 °C for 48 h. Only bags recovered within 24 h after insertion were used for further analysis.

### 2.5. Chemical Analysis

Forage samples and incubated residuals were dried in an air oven (DGG-9240B; Shanghai-ShenXin Inc, Shanghai, China) at 65 °C for 48 h to determine DM content. To ensure a homogeneous mixture, all samples were ground through a mill equipped with a 1 mm screen (KRT-34; KunJie, Beijing, China). Starch (method 996.11), nitrogen (method 984.13), ether extract (method 920.39), and ash (method 924.05) were determined according to the methods described by the Association of Official Analytical Chemists (AOAC) [56]. The contents of NDF and ADF were analyzed by the Ankom fiber analyzer (A2000i; Ankom Technology, Fairport, NY, USA) following the procedures of Van Soest et al. [57]. The content of acid detergent lignin (ADL) was determined by the solubilization of cellulose with 72% sulphuric acid. Hemicellulose and cellulose were then calculated as the differences between NDF and ADF, ADF, and ADL, respectively [51]. All forage samples and fermentation residues were conducted in triplicate.

### 2.6. Calculations

The degradation kinetics of CP, NDF, and ADF from nylon bags were calculated according to the following exponential equation [58]:

(1) $$y=a+b\;\left({1\;-\;e\;}^{-c\;t}\right)$$

where y is the ruminal degradation of DM, CP, NDF, and ADF at time t, a is the rapidly degradable fraction (g/kg). b is the potentially degradable fraction (g/kg), c is the constant rate of degradation of b (%/h), and t is the time of incubation (h). The calculated potential degradable fraction (cpd, g/kg) was calculated as a + b.

The effective degradability (ED) of nutrients was calculated according to the following equation [58]:

(2) $$\mathrm{ED}=a+\left(\frac{\mathrm{bc}}{c+\mathrm{kp}}\right)$$

where a, b, and c are the same parameters represented in Equation (1) and k (%/h) is the rumen particle passage rate. The NRC (2001) [51] gives the rumen passage rate (kp) prediction equation for forage as follow:

(3) $$\mathrm{kp}=3.362+0{.479\;\times \;X}_{1}-\;0{.007\;\times \;X}_{2}-\;0{.017\;\times \;X}_{3}$$

where X_1_, X_2_, and X_3_ are dry matter intake (% of body weight), percentage of concentrate in the diet DM, and percentage of NDF in DM, respectively. Therefore, the kp value of 4.18%/h was calculated according to NRC (2001) (Equation (3)) [51] with dry matter intake of 23.68 kg/d, diet forage-to-concentrate ratio of 61.1: 38.9, and NDF concentration of 29.7% in DM diet.

The indigestible neutral detergent fiber (iNDF) were determined by long-term (288 h) in situ ruminal incubation, and the iNDF content is calculated according to the following equation [17]:

(4) $${\mathrm{iNDF}}_{288}\;(g/\mathrm{kg}\;\mathrm{of}\;\mathrm{NDF})=(\frac{{\mathrm{NDF}}_{288}}{\mathrm{NDF}})\;\times \;1000$$

where iNDF_288_ (g/kg of NDF) is the total indigestible NDF fraction of the forage NDF; NDF_288_ (g/kg) is the amount of NDF in the bag remaining after 288 h of ruminal incubation; NDF (g/kg) is the amount of NDF in the bag before ruminal incubation. The determined potentially digestible NDF (dpdNDF) were measured according to the report of Rinne et al. [17] as follows:

(5) $${\mathrm{dpdNDF}}_{288}=\mathrm{NDF}-{\;\mathrm{iNDF}}_{288}$$

In the present study, the dpdDM_288_, dpdCP_288_, and dpdADF_288_ were calculated according to the above-modified equation for dpdNDF_288_. The intestinal digestibility (Idg, %) of ruminal undegradable nutrients in the residuals was calculated according to the following equation:

(6) $$\mathrm{Idg}=\frac{(C1\;\times \;W1\;-\;C2\;\times \;W2)}{C1\;\times \;W1}\times 100$$

where C1, W1, C2, and W2 are the nutrients in the undegradable residue after 12 h (CP), 24 h (NDF and ADF) ruminal incubation (g/kg), the weight of undegradable residue placed into the mobile nylon bag for intestine incubation (g), the nutrients content in the residue (g/kg), and the weight of intestinal undigestible residue (g).

The intestinal digestible CP (ID_CP_, g/kg of CP in initial forage) of ruminal undegradable CP was calculated using the following modified equation from the Dutch protein evaluation system DVE/OEB_2007_ [59,60]:

(7) $${\mathrm{ID}}_{\mathrm{CP}}=\mathrm{CP}\;\times \;\frac{\mathrm{RUP}}{100}\;\times \frac{\mathrm{Idg}}{100}$$

where CP (g/kg) is the CP content in initial forage; RUP (g/kg of CP) is the ruminal undegradable protein in incubated residue; and Idg (%) is represented in Equation (7). RUP is the ruminal undegradable CP content after 12 h ruminal incubation. In the present study, the ID_NDF_ and ID_ADF_ were calculated according to the above-modified equation for ID_CP_.

Total-tract neutral detergent fiber digestibility (TTNDFD; g/kg of NDF) was calculated according to the study of Lopes et al. [26].

(8) TTNDFD=(ruminal NDF degradation)+(intestinal NDF digestion)

where ruminal NDF degradation (g/kg of NDF) is the ruminal degradable NDF content after 24 h incubation, intestinal NDF digestion (g/kg of NDF) is the intestinal digestible NDF content. In the present study, the TTD_CP_ and TTD_ADF_ were calculated according to the above-modified equation for TTNDFD.

### 2.7. Statistical Analysis

All data were analyzed using SAS (version 9.4, SAS Institute Inc., Cary, NC, USA). The ruminal degradation kinetics (i.e., a, b, c, cpd, dpd, and ED) of CP, NDF, and ADF within various forages were estimated using the NLIN procedure (Equations (1) and (2)). Data for ruminal degradability, intestinal digestibility, and total tract digestibility of CP, NDF, and ADF within various forages were summarized by descriptive statistics and analyzed using the MIXED procedure of SAS based on the following model:

(9) Yij = μ + Fi + rj + eij 

where Yijk was the dependent variable, μ was the overall mean, Fi was the fixed effect of forage (i = 1–5), rj was the random effect of replicate (j = 1–6), and eij = the residual error. Six replicates were used in the ruminal degradation experiment. Intestinal digestibility tests were conducted with 12 replicates for each forage. Statistical differences were considered significant at *p* < 0.05. The simple linear regression equations were derived between the dpd_288_ (determined potential degradation fraction by 288 h ruminal incubation, Equation (5)) and cpd (calculated potential degradation fraction, Equation (1)). The multiple linear regression equations were estimated by the PROC CORR and REG procedures in SAS 9.4 to predict iNDF_288_ and TTD fractions from the chemical composition and ruminal degradation kinetics of different forages.

**Figure 1:**
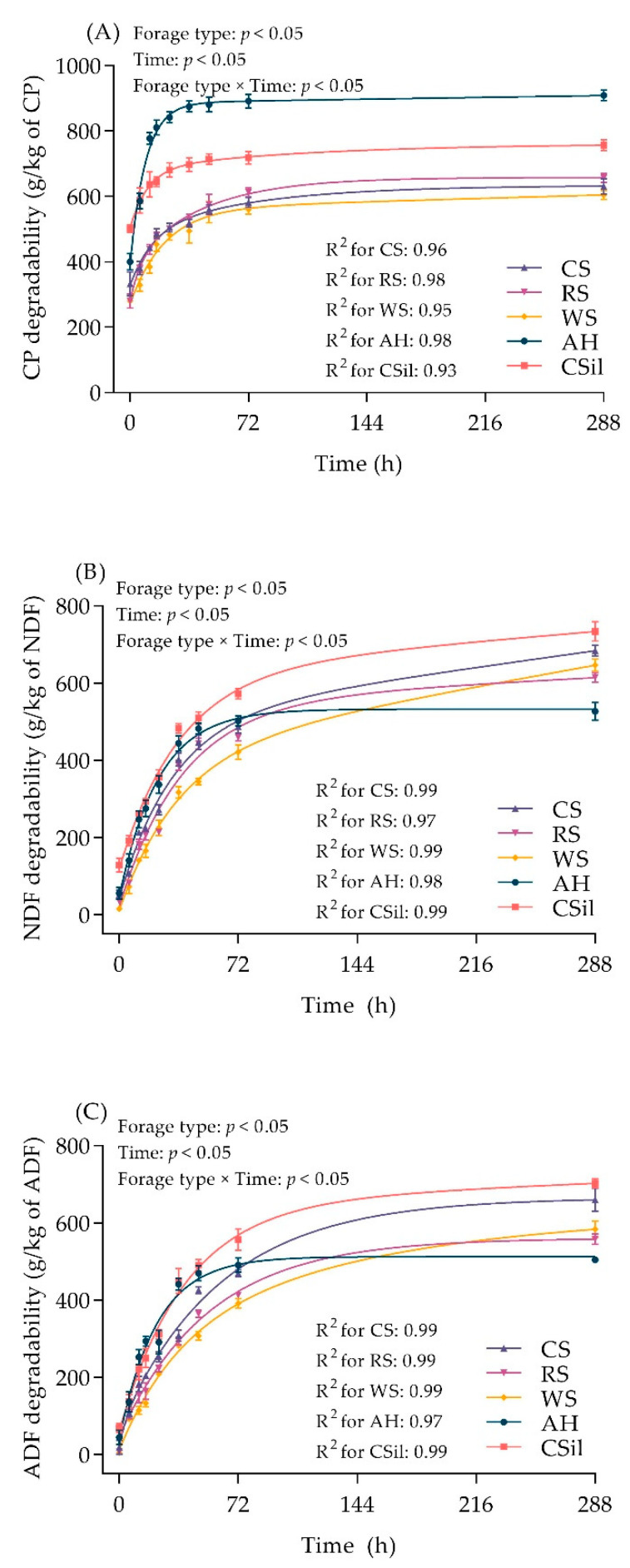
The real-time degradability of various forages during 288 h in situ incubation. (**A**) CP, (**B**) NDF, and (**C**) ADF. Data are expressed as means ± standard deviation. *p*-values of Forage Type, Time, and Forage Type × Time mean the effect of forage types, the effect of incubation times, and the interaction effect of forage types and incubation times. Data were fitted to a one-phase association exponential model with three parameters: y = a + b × [1 − exp (−c × t)], where y is the ruminal degradation of CP, NDF, and ADF at time t, a is the soluble or rapidly degradable fraction (g/kg); b is the potentially degradable fraction (g/kg); c is the constant rate of degradation of b (%/h); t is the time of incubation (h). Regression, *p <* 0.05; R^2^ for CS, RS, WS, AH, and CSil = 0.93 to 0.99, as shown in the graph. CS, corn straw; RS, rice straw; WS, wheat straw; AH, alfalfa hay; Csil, corn silage; DM, dry matter; CP, crude protein; NDF, neutral detergent fiber; ADF, acid detergent fiber. Each forage was incubated in 6 replicates (two for each time point per cow) in the rumen.

All figures were performed using GraphPad Prism (version 9.0.1, GraphPad, San Diego, CA, USA). Plotting and curve-fitting of data (Figure 1) were fitted to a one-phase association exponential model with three parameters: y = a + b × [1 − exp (−c × t)] (Equation (1)) in GraphPad Prism. Data are expressed as means ± standard deviation in the figure. Effects of forage type and incubation time onCP, NDF, and ADF degradability variables were analyzed using the MIXED procedure of SAS according to the following equation:

(10) Yijk = μ + Fi + rj + Tj + FTij + eijk 

where Yijk was the dependent variable, μ was the overall mean, Fi was the fixed effect of forage (i = 1–5), rj was the random effect of replicate (j = 1–6), Tj was the incubation time effect (j = 1–9), FTij was the interaction between the forage types and incubation time, and eij was the residual error.

## 3. Results

### 3.1. In Situ Ruminal Degradability and iNDF_288_

The chemical compositions of the individual forage are listed in Table 1. Compared with AH and CSil, CS, RS, and WS had lower amounts of CP, ether extract, and NFC, and greater amounts of NDICP, ADICP, NDF, ADF, hemicellulose, and cellulose. The tropical (C4) forages (CS and CSil) had lower amounts of ADL (g/kg of DM), ADL (g/kg of NDF), and iNDF_2.4_ (g/kg of NDF) compared to those of subtropical (C3) forages (RS, WS, and AH). AH had the highest iNDF_2.4_ and iNDF_288_ values of 379.42 and 473.40 g/kg of NDF. CS and CSil had similar and lower iNDF_2.4_ values of 177.44 and 179.43 g/kg of NDF, while CSil had the lowest iNDF_288_ value of 265.92 g/kg of NDF.

The real-time degradability of CP (A), NDF (B), and ADF (C) within various forages increased during 288 h in situ incubation (Figure 1), and the nonlinear model resulted in a high average coefficient of determination (R^2^ > 0.93) for all forages. There were significant differences in CP, NDF, and ADF degradability among the five forages during the entire 288 h incubation period (*p* value for the interaction effect of forage types and incubation times <0.05). The CP degradability of CS, RS, and WS were lower than AH and Csil during the entire incubation period. Table 2 outlines ruminal degradation kinetics and effective degradability of CP, NDF, and ADF of various forages. CS, RS, and WS had lower a, cpd, dpd_288_, and ED of CP compared to AH and CSil (*p* < 0.05). The cpd and dpd of CSil were highest, with the CS, RS, and WS being intermediate, and AH the lowest (*p* < 0.05). Lower ED of CP, NDF, and ADF was observed in CS, RS, and WS compared with AH and CSil (*p* < 0.05).

### 3.2. Intestinal and Total-Tract Digestibility

**Figure 2:**
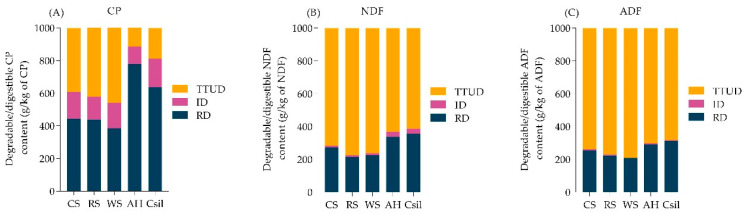
Ruminal degradable content, intestinal digestible content, and total-tract undigested content of CP, and NDF, and ADF in different forages. (**A**) CP, (**B**) NDF, and (**C**) ADF. RD, ruminal degradable content was determined after ruminal incubation for 12 h (CP) and 24 h (NDF and ADF); ID, intestinal digestible content; TTUD, total-tract undigested content. CS, corn straw; RS, rice straw; WS, wheat straw; AH, alfalfa hay; Csil, corn silage; DM, dry matter; CP, crude protein; NDF, neutral detergent fiber; ADF, acid detergent fiber. Each forage was incubated in 6 replicates (two for each time point per cow) in the rumen and 12 replicates for intestinal incubation.

Intestinal digestible, and total-tract digested content of CP, NDF, and ADF in different forages are listed in Table 3. Compared with AH and CSil, CS, RS, and WS had lower total-tract digestibility of CP, NDF, and ADF (*p* < 0.05), lower intestinal digestibility of rumen undegradable content of NDF (*p* < 0.05). Meanwhile, the tropical (C4) forages (CS and CSil) had lower ID_CP_ compared to subtropical (C3) forages (RS, WS, and AH) (*p* < 0.05). The ruminal degradable, intestinal digestible, and total-tract undigested content of CP, NDF, and ADF for all the forages used in this experiment are shown in Figure 2. Larger amounts of CP were digested compared to NDF and ADF in the intestine.

### 3.3. Prediction of iNDF_288_ and Total-Tract Digestibility

The multiple linear regression equations for the prediction of iNDF_288_ and TTD based on chemical composition and ruminal degradation kinetics are presented in Table 4. Regression analysis showed that the iNDF_288_ was influenced by the ADL ((g/kg of NDF) content in the forage samples (R^2^ = 0.995). TTD_NDF_ and TTD_ADF_ were jointly influenced by the NDF and ADF content, while TTD_CP_ were jointly influenced by the CP, NDF, and ADF content in the forage samples.

## 4. Discussions

Digestibility in ruminants is affected by feed type, chemical composition, animal DM intake, healthy status, rumen bacteria [52,61,62]. Different forages vary in their chemical composition, resulting in different digestibility and the efficiency of energy utilization in dairy cows [16,18,63]. The digestibility and feed values of CS, RS, and WS were reported to be low due to their chemical composition, which may restrict their utilization by ruminants as a forage resource [11,12,13]. Wang et al. reported that corn stover and rice straw had lower amounts of CP (5.9 and 5.5 vs. 17.4 and 8.1% of DM) and NFC (11.6 and 5.2 vs. 22.4 and 14.6% of DM), as well as greater amounts of NDF (74.1 and 74.5 vs. 51.4 and 69.5% of DM) and ADF (39.7 and 45.5 vs. 37.0 and 34.0% of DM), compared to AH and CSil [14]. Sarnklong et al. published the mean values of N content, NDF, ADF, hemicellulose, cellulose, and ADL of rice straw as 0.96, 73.01, 41.59, 31.42, and 4.84% of DM, respectively [11]. Consistently, the CS, RS, and WS in the current study had lower amounts of CP and NFC, and greater NDF, ADF, hemicellulose, and cellulose contents compared to those of AH and CSil. Meanwhile, the contents of NDF (420.67 g/kg of DM) and ADF (246.23 g/kg of DM) of Csil in the current study were lower compared to the results published by Wang et al. [14], which might be due to the differences in the stage of maturity and the grain content.

The rates of pdNDF degradation and effective degradability of NDF for AH and CSil in the present study were consistent with those from previous studies [18,43,49]. The constant rate of degradation of pdNDF and ED of NDF for CSil (*n* = 74) ranged from 1.23 to 3.17%/h and from 36.5 to 61.4%, respectively [43]. Rates of pdNDF digestion ranged from 0.0426 to 0.0569/h and from 0.1402 to 0.0515/h for maize silage (*n* = 17) and Lucerne (*n* = 10), respectively [18]. The CP content in forage has been reported to be the most limiting nutrient parameter when the CP was below the lowest threshold level (8.0%) [64]. The natural pasture hay had very low CP content (38.8 g/kg DM), which was below the CP requirements for ruminant animals for proper rumen function and efficient microbial activity [64,65]. Unsurprisingly, the CP, NDF, and ADF degradability of CS, RS, and WS were lower than those of AH and Csil during the first 48 h of incubation, implying the low nutritional value of crop straws. A 1-percentage-unit change in NDF digestibility (NDFD) has been correlated with a 0.17 kg increase in voluntary DMI and a 0.25 kg increase in 4% FCM yield [16]. However, the NDF and ADF degradability of the crop straws exceeded those of AH at 288 h of incubation, indicating that crop straws have a larger amount of potential degradation fraction.

The iNDF has been demonstrated to be a good predictor of forage digestibility and is an important parameter in mechanistic rumen models [17,19,20,21,22]. The CNCPS [24] and CPM Dairy [25] had previously used a factor iNDF_2.4_ to describe the iNDF of forages. It should be noted that the tropical (C4) forages (CS and CSil) had lower amounts of ADL and iNDF_2.4_ compared to those of subtropical (C3) forages (RS, WS, and AH). Furthermore, it is remarkable that AH had the highest iNDF_2.4_ and iNDF_288_ values (379.42 and 473.40 g/kg of NDF), and CSil had the lowest iNDF_288_ value (265.92 g/kg of NDF) in the present study. Consistently, Raffrenato et al. analyzed more than two hundred samples of several forage species from Australia and South Africa; the results indicated that the lignin and iNDF were highest in legumes and C3 forages on NDF basis [48]. These observations already demonstrate how the plant species and growing conditions play an important role in determining the chemical and structural relationship between the indigestible cell wall components and iNDF content. Therefore, ADL seems to have a more negative effect in determining iNDF in forages. The iNDF/ADL ratios in the present study were 4.27, 4.28, 4.03, 2.99, and 3.56 for CS, RS, WS, AH, and CSil, respectively. While AH resulted in ratios closer to 2.4, and different environmental conditions caused higher ratios for alfalfa samples, other forages averaged around 4 during the same time [48,49,66], which was consistent with the present study. These observations demonstrate that the value of 2.4 cannot be valid among all forages, and it represents higher values for iNDF_288_ compared to iNDF_2.4_ across all forage samples. Using iNDF_240_ (ruminal incubation for 240 h) showed consistently lower ME between 2 and 10 MJ/day, compared to when using iNDF_2.4_. As a consequence, the improved metabolizable protein and ME values would result in 0.3 to 3.2 kg/d less (10% reduction) milk, compared to when using iNDF2.4 [49].

TTD has been demonstrated to be a good predictor of feed digestibility [27]. The intestinal digestible CP among all forages in the present study ranged from 108.76–175.22 g/kg of CP. Similar results in sub-irrigated meadow and upland native range grass were reported by Buckner et al. [67]. However, higher intestinal digestible CP with average values of 0.353 in maize silages and 0.237 in grass silages were reported by Ali et al. [47]. This might be due to differences in forage species, forage CP content, and the ruminal degradable fraction in the two studies. TTD of CP, NDF, and ADF in the present study were 541.52–886.94 g/kg of CP, 226.35–387.28 g/kg of NDF, and 209.12–317.69 g/kg of ADF, respectively. The contribution in the post ruminal digestion of the TTNDFD is low, as cows do not secrete enzymes with fibrinolytic activity; rumen undegradable NDF and ADF cannot be digested in the small intestines, but might make up 0 to 0.20 of TTNDFD fermented in the hindgut [47]. Therefore, a longer ruminal incubation period would result in a greater amount of TTNDFD. Nevertheless, similar TTNDFD results of Csil and AH were reported in previous studies [26,47,68]. Lower TTNDFD in crop straws were determined in the current study, as the crop straws consist predominantly of the cell wall, which is made up of cellulose, hemicellulose, and lignin.

The regression equations showed that the iNDF_288_ and TTD were influenced by the chemical composition of forages, especially ADL, NDF, ADF, ash, and CP. Although only five forages were included in the equation, a general regression equation with a satisfactory accuracy (R^2^ = 0.995; RMSE = 7.51 g/kg of NDF) was derived for the prediction of iNDF_288_ based on ADL and ash contents of forages. Contrarily, Raffrenato et al. analyzed more than two hundred samples of several forage species from Australia and South Africa, and similar equations for the prediction of iNDF were determined [48]. Lopes et al. noted that a good model to predict TTNDFD should consider more parameters, such as iNDF, pdNDF and kp [68]. High accuracies of regression equations (R^2^ ≥ 0.918) for TTD prediction were generated based on NDF, ADF, and CP content of forages. A more accurate and precise estimation of iNDF and TTD would significantly improve the fine-tuning of dairy cow diets, especially when using high forage and/or NDF rations.

## 5. Conclusions

Generally, CS, RS, and WS had lower nutritional values compared to AH and Csil. However, CS, RS, and WS had higher ruminal potential NDF degradation, intestinal digestible CP, and lower iNDF_288_ content compared to AH. Incorporating this information into rations could improve our ability to optimize the utilization of main crop straws and milk production. Equations based on chemical compositions and ruminal degradation kinetics can give acceptable estimates of iNDF_288_ and TTD.

## Figures and Tables

**Table 1:** Chemical composition and indigestible NDF of the forage samples (g/kg of DM, unless otherwise indicated).

Item ^1^	CS	RS	WS	AH	CSil
DM (g/kg of fresh matter)	911.13	929.65	919.97	911.23	316.23
OM	915.73	875.03	902.17	896.47	944.83
CP	55.23	43.73	44.37	203.90	89.87
NDICP (g/kg of CP)	258.78	227.36	253.34	272.43	174.24
ADICP (g/kg of CP)	141.02	117.57	138.4	58.75	67.82
Starch	27.84	23.80	22.93	11.50	309.37
Ether extract	14.33	16.77	13.45	21.94	34.93
NFC ^2^	109.70	99.76	29.67	230.50	339.36
NDF	736.47	714.77	814.68	440.13	420.67
ADF	431.31	454.90	514.19	316.10	246.23
Hemicellulose	305.16	259.87	300.49	124.03	174.44
Cellulose	376.86	390.52	442.72	246.52	214.78
ADL ^3^	54.45	64.38	71.47	69.58	31.45
ADL (g/kg of NDF)	73.93	90.07	87.73	158.09	74.76
iNDF_2.4_ ^4^ (g/kg of NDF)	177.44	216.17	210.55	379.42	179.43
iNDF_288_ ^5^ (g/kg of NDF)	315.64	385.07	353.61	473.40	265.92
iNDF_288_/ADL ^6^	4.27	4.28	4.03	2.99	3.56

^1^ CS, corn straw; RS, rice straw; WS, wheat straw; AH, alfalfa hay; Csil, corn silage; DM, dry matter; OM, organic matter; CP, crude protein; NDICP, neutral detergent-insoluble crude protein; ADICP, acid detergent-insoluble crude protein; NFC, non-fiber carbohydrate; NDF, neutral detergent fiber; ADF, acid detergent fiber. ^2^ NFC (g/kg of DM) = 1000 − (CP + NDF + EE + Ash). ^3^ ADL: acid detergent lignin, determined by cellulose solubilization with sulphuric acid. ^4^ iNDF_2.4_, calculated as ADL (g/kg of NDF) × 2.4. ^5^ iNDF_288_, indigestible NDF determined by ruminal incubation for 288 h (Equation (5)). ^6^ iNDF/ADL, iNDF_288_ (g/kg of NDF)/ADL (g/kg of NDF). Values of chemical composition and iNDF_2.4_ represent the means of 3 parallel determinations or calculations, values of iNDF_288_ represent the means of 6 replicates.

**Table 2:** In situ ruminal degradation kinetics and effective degradability of CP, NDF, and ADF in different forages (*n* = 6).

Item ^1^	CS	RS	WS	AH	CSil	SEM ^2^	*p* Value
CP (g/kg of CP, unless otherwise indicated)							
a ^3^	332.91 ^bc^	294.98 ^cd^	270.04 ^d^	364.25 ^b^	501.86 ^a^	22.37	<0.05
b ^3^	284.16 ^cd^	344.21 ^b^	321.75 ^bc^	529.95 ^a^	231.92 ^d^	27.91	<0.05
c ^3^ *(*%/h)	3.94 ^c^	3.94 ^c^	4.19 ^c^	10.81 ^a^	6.89 ^b^	0.80	<0.05
cpd_CP_ ^3^	617.08 ^cd^	639.19 ^c^	591.80 ^d^	894.20 ^a^	733.78 ^b^	29.83	<0.05
dpd_CP288_ ^4^	631.53 ^cd^	656.76 ^c^	604.98 ^d^	909.72 ^a^	757.06 ^b^	30.02	<0.05
ED_CP4.18_ ^5^	466.24 ^c^	461.69 ^c^	429.92 ^d^	746.60 ^a^	642.34 ^b^	33.14	<0.05
NDF (g/kg of NDF, unless otherwise indicated)							
a ^3^	53.09 ^b^	22.53 ^c^	23.51 ^c^	38.06 ^c^	123.06 ^a^	10.11	<0.05
b ^3^	623.53 ^a^	587.52 ^b^	623.89 ^a^	493.23 ^c^	605.80 ^ab^	13.46	<0.05
c ^3^ *(*%/h)	1.99 ^b^	2.27 ^b^	1.57 ^c^	4.31 ^a^	2.15 ^b^	0.26	<0.05
cpd_NDF_ ^3^	676.63 ^b^	610.05 ^c^	647.40 ^b^	531.28 ^d^	728.85 ^a^	18.20	<0.05
dpd_NDF288_ ^4^	684.36 ^b^	614.93 ^c^	646.39 ^c^	526.60 ^d^	734.08 ^a^	19.09	<0.05
ED_NDF4.18_ ^5^	253.83 ^c^	229.08 ^d^	193.54 ^e^	288.31 ^b^	327.78 ^a^	12.54	<0.05
ADF (g/kg of ADF, unless otherwise indicated)							
a ^3^	37.40 ^b^	24.67 ^b^	19.30 ^b^	32.58 ^b^	59.02 ^a^	4.19	<0.05
b ^3^	621.37 ^a^	530.65 ^b^	567.40 ^b^	478.73 ^c^	639.54 ^a^	16.64	<0.05
c ^3^ *(*%/h)	1.82 ^c^	2.01 ^bc^	1.57 ^c^	4.51 ^a^	2.31 ^b^	0.29	<0.05
cpd_ADF_ ^3^	658.78 ^b^	555.31 ^c^	586.70 ^c^	511.32 ^e^	698.55 ^a^	18.81	<0.05
dpd_ADF288_ ^4^	660.06 ^b^	558.26 ^c^	583.39 ^c^	504.50 ^d^	702.92 ^a^	19.43	<0.05
ED_ADF4.18_ ^5^	225.31 ^b^	196.57 ^c^	173.97 ^d^	280.82 ^a^	286.22 ^a^	12.07	<0.05

^a–e^ Means with different lowercase superscript letters within rows represent significant differences at *p*-value < 0.05. ^1^ CS, corn straw; RS, rice straw; WS, wheat straw; AH, alfalfa hay; Csil, corn silage; DM, dry matter; CP, crude protein; NDF, neutral detergent fiber; ADF, acid detergent fiber. ^2^ SEM, standard error of the mean. ^3^ a, the rapid degradable fraction; b, the slow degradable fraction; c, the constant rate of degradation of b (%/h); cpd, the calculated potential degradable fraction, calculated as a + b. ^4^ dpd, the determined potential degradable fraction, the degradable fraction after 288 h ruminal incubation. ^5^ ED, effective degradability, values with different capital subscript letters (CP, NDF, and ADF).

**Table 3:** Ruminal degradable, intestinal digestible, and total-tract digested content of CP, NDF, and ADF in different forages (*n* = 12).

Item ^1^	CS	RS	WS	AH	Csil	SEM ^2^	*p* Value
CP (g/kg of CP, unless otherwise indicated)							
RUD_CP_ ^4^ (12 h)	556.48 ^b^	562.72 ^b^	615.33 ^a^	221.82 ^d^	362.56 ^c^	39.94	<0.05
Idg of RUD_CP_ ^5^ (%)	29.75 ^b^	25.49 ^b^	25.19 ^b^	49.03 ^a^	48.33 ^a^	3.11	<0.05
ID_CP_ ^6^	165.55 ^a^	141.57 ^b^	156.85 ^ab^	108.76 ^c^	175.22 ^a^	6.56	<0.05
TTD_CP_ ^7^	609.08 ^c^	579.57 ^d^	541.52 ^e^	886.94 ^a^	812.67 ^b^	36.86	<0.05
TTUD_CP_ ^8^	390.92 ^c^	420.43 ^b^	458.48 ^a^	113.06 ^e^	187.33 ^d^	36.86	<0.05
NDF (g/kg of NDF, unless otherwise indicated)							
RUD_NDF_ ^4^ (24 h)	727.77 ^b^	785.27 ^a^	774.83 ^a^	662.83 ^c^	643.55 ^c^	15.79	<0.05
Idg of RUD_NDF_ ^5^ (%)	1.54 ^b^	1.48 ^b^	1.63 ^b^	4.73 ^a^	4.79 ^a^	0.69	<0.05
ID_NDF_ ^6^	11.21 ^b^	11.62 ^b^	12.63 ^b^	31.35 ^a^	30.83 ^a^	2.53	<0.05
TTD_NDF_ ^7^	283.44 ^b^	226.35 ^c^	237.8 ^c^	368.52 ^a^	387.28 ^a^	18.06	<0.05
TTUD_NDF_ ^8^	716.56 ^b^	773.65 ^a^	762.20 ^a^	631.48 ^c^	612.72 ^c^	18.06	<0.05
ADF (g/kg of ADF, unless otherwise indicated)							
RUD_ADF_ ^4^ (24 h)	746.49 ^b^	776.72 ^a^	790.88 ^a^	708.85 ^c^	688.65 ^c^	11.00	<0.05
Idg of RUD_ADF_ ^5^ (%)	1.10	0.83	−0.07	0.98	0.92	0.77	0.947
ID_ADF_ ^6^	8.21	6.45	−0.55	6.95	6.34	0.71	0.938
TTD_ADF_ ^7^	261.72 ^b^	229.73 ^c^	209.12 ^c^	298.10 ^a^	317.69 ^a^	11.38	<0.05
TTUD_ADF_ ^8^	738.28 ^b^	770.27 ^a^	790.88 ^a^	701.90 ^c^	682.31 ^c^	11.38	<0.05

^a^^–e^ Means with different lowercase superscript letters within rows represent the significant differences at *p*-value < 0.05. ^1^ CS, corn straw; RS, rice straw; WS, wheat straw; AH, alfalfa hay; Csil, corn silage; DM, dry matter; CP, crude protein; NDF, neutral detergent fiber; ADF, acid detergent fiber. ^2^ SEM, standard error of the mean. ^3^ RD, ruminal degradable content which determined by ruminal incubation for 12 h (RDP) and 24 h (RD_NDF_ and RD_ADF_). ^4^ RUD, ruminal degradable content which determined by ruminal incubation for 12 h (RUP) and 24 h (RUD_NDF_ and RUD_ADF_). ^5^ Idg (%), intestinal tract digestibility of rumen undegradable content. ^6^ ID, intestinal digestible content of rumen degradable content. ^7^ TTD, total-tract digested content. ^8^ TTUD, total-tract undigested content.

**Table 4:** The multiple linear regression equations for predicting iNDF_288_ and total-tract digestibility based on chemical composition and ruminal degradation kinetics of different forages (*n* = 5).

Regression Equation ^1^	RMSE ^2^	R^2^ Value ^3^
iNDF_288_		
iNDF_288_ = 74.52 + 1.64 ADL ** + 1.35 Ash **	7.51	0.995
iNDF_288_ = 0.85 iNDF_2.4_ ** + 162.07	37.39	0.828
dpd_NDF288_ = 1.05 cpd_NDF_ ** − 30.01	3.98	0.998
Total-tract digestibility (TTD)		
TTD_CP_ = 922.67 + 1.13 CP ** − 0.10 NDF * − 0.68 ADF **	9.49	0.999
TTD_NDF_ = 554.41 − 0.10 NDF * − 0.49 ADF **	29.97	0.918
TTD_ADF_ = 424.19 + 0.06 NDF * − 0.50 ADF **	12.38	0.963

** represents the variables included in the multiple linear regressions were significant differences at *p*-value < 0.01, * represents the significant differences at *p*-value < 0.05;^1^ iNDF_288_, indigestible NDF determined by ruminal incubation for 288 h (Equation (4)). ADL, acid detergent lignin (g/kg of NDF), determined by solubilization of cellulose with sulphuric acid. iNDF_2.4_, calculated as ADL (g/kg of NDF) × 2.4. dpd_NDF288_, the determined potential degradation NDF (g/kg of NDF), thedegradable NDF after 288 h ruminal incubation, iNDF_288_ could also be derived according to NDF—dpd_NDF288_. cpd, the calculated potential degradation fraction, calculated as a + b. TTD, total-tract digested content, calculated as ruminal degradable content + intestinal digestible content. CP, crude protein; NDF, neutral detergent fiber; ADF, acid detergent fiber. ^2^ RMSE, root mean square error of intercepts in the linear regressions. R^2^ value (R-Squared), is a statistical measure of fit that indicates how much variation of a dependent variable is explained by the independent variables in a regression model.
